# *Lactobacillus plantarum* KSFY06 on d‐galactose‐induced oxidation and aging in Kunming mice

**DOI:** 10.1002/fsn3.1318

**Published:** 2019-12-05

**Authors:** Fang Li, Guangbin Huang, Fang Tan, Ruokun Yi, Xianrong Zhou, Jianfei Mu, Xin Zhao

**Affiliations:** ^1^ Chongqing Collaborative Innovation Center for Functional Food Chongqing University of Education Chongqing China; ^2^ Chongqing Engineering Research Center of Functional Food Chongqing University of Education Chongqing China; ^3^ Chongqing Engineering Laboratory for Research and Development of Functional Food Chongqing University of Education Chongqing China; ^4^ College of Biological and Chemical Engineering Chongqing University of Education Chongqing China; ^5^ Department of Trauma Surgery Emergency Medical Center of Chongqing The Affiliated Central Hospital of Chongqing University Chongqing China; ^6^ Department of Public Health Our Lady of Fatima University Valenzuela Philippines

**Keywords:** aging, *Lactobacillus plantarum* KSFY06, mice, oxidation

## Abstract

Yogurt from Xinjiang, China, is a traditional Chinese fermented food rich in beneficial microorganisms, such as *Lactobacillus plantarum* KSFY06. In this study, the effect of KSFY06 on oxidative aging was investigated using live animal experiments. Molecular biological methods were used to analyze the serum and tissues of mice with oxidative aging induced by d‐galactose, which showed that KSFY06 can inhibit the decline of heart, liver, spleen, and kidney caused by aging. The KSFY06 strain increased the activity of superoxide dismutase (SOD), glutathione peroxidase (GSH‐Px), catalase (CAT), and glutathione (GSH) in serum and liver of aging mice, while the content of malondialdehyde (MDA) is reduced. Pathological observation showed that KSFY06 alleviated damage to the liver, spleen, and skin of oxidative aging mice. qPCR showed that, at high dose (2 × 10^9^ cfu/kg per day), KSFY06 upregulates copper/zinc superoxide dismutase (SOD1), manganese superoxide dismutase (SOD2), endothelial nitric oxide synthase (eNOS), neuronal nitric oxide synthase (nNOS), catalase (CAT) mRNA expression, and its downstream inducible nitric oxide synthase (iNOS) mRNA expression in liver and spleen tissues induced by d‐gal. To a certain extent, these findings indicate that *L. plantarum* KSFY06 is able to protect against oxidative stress in the d‐gal‐induced aging model. In conclusion, *L. plantarum* KSFY06 may provide a potential research value in the prevention or alleviation of related diseases caused by oxidative stress.

## Graphical abstract

This study explored the effect of KSFY06 on oxidative aging in vivo. KSFY06 may provide a promising dietary approach for the prevention or alleviation of oxidative stress and age‐related conditions.

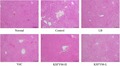

## 1 INTRODUCTION

Xinjiang is located in the northwest border of China. Animal husbandry is one of the most distinctive traditional basic industries in Xinjiang. Many minority herdsmen are accustomed to producing various dairy products by natural fermentation (Ren, Li, & Guo, 2016). The most popular one is yogurt, which contains a variety of lactic acid, lactose, vitamins, and enzymes while exhibiting a considerably higher nutritional value than fresh cow or sheep milk (Li, Mutuvulla, Chen, Jiang, & Dong, 2012). The protein contained in Xinjiang yogurt has a higher physiological value than rice, white flour, and meat (Zhao et al., 2019). *Lactobacillus* in yogurt can inhibit and eliminate spoilage bacteria such as typhoid bacillus, dysentery bacillus, and staphylococcus in the intestine, thereby helping to improve the distribution of intestinal flora, inhibit the production of poisonous bacteria, enhance intestinal function, and promote nutrition (Li, Wang, Liu, Guo, & Li, 2013). The Xinjiang Uygur people, whose diet is rich in the local yogurt, have less cancer, suggesting Xinjiang yogurt also has an antioxidation and anticancer effect (He et al., 2015). Lactic acid bacteria (LAB) are the most commonly used microorganisms in fermented foods (Chen et al., 2018, 2017). Lactic acid bacteria play an important role in yogurt because the lactic acid produced by these bacteria can be used as natural preservatives and flavor enhancers (Li et al., 2012).

Probiotics including LAB and bifidobacteria are the most studied microorganisms in recent decades. Probiotics are unique in that they participate in food fermentation and provide a variety of nutrients needed by the human body. Tolerance to acid and bile conditions, ability to reduce cholesterol, ability to hydrolyze bile salts, nonhemolysis, ability to resist microorganisms, and ability to survive during fermentation are all criteria for screening probiotics (Kumar, Vijayendra, & Reddy, 2015). The probiotics we commonly use are isolated from yogurt. Probiotics are more and more accepted because they can stimulate the host immune system, reduce the risk of cancer, and resist hypertension, antioxidant, cholesterol, and other functions by preventing nosocomial microbial infection (Abushelaibi, Al‐Mahadin, El‐Tarabily, Shah, & Ayyash, 2017; Li et al., 2012; Linares, Martín, Ladero, Alvarez, & Fernández, 2011).

As the human lifespan increases, aging is inevitable, the result of stress and strain, injury and infection, impaired immune response, malnutrition, metabolic disorders, and accumulation of negligent and abusive drugs (Covinsky et al., 2003; Zhou et al., 2017). Aging is associated with diseases such as hypertension, type 2 diabetes, and atherosclerosis. Delaying aging can delay the onset of these diseases (Badgujar, Chandratre, Pawar, Telang, & Kurade, 2016). d‐galactose is considered to be a drug that can control aging (Suo et al., 2018). d‐galactose‐induced aging model is similar to natural aging and has been used in disease treatment. The reason of aging in this model is that intracellular galactose is reduced to galactitol and cannot be further degraded. Accumulated galactitol leads to metabolic disorders in cells, producing reactive oxygen species (ROS), which induces aging (Ho, Liu, & Wu, 2003; Zhao et al., 2017). d‐galactose‐induced oxidative stress is often prevented by probiotics such as Lactobacillus and Bifidobacterium. Therefore, probiotics are also used in the development of antioxidants and health products (Wang, Xie, et al., 2016; Wang, Zhou, Xia, Zhao, & Shao, 2016; Yu et al., 2016).

In this study, a new strain of LAB was isolated from yogurt from Xinjiang, China, and named *Lactobacillus plantarum* KSFY06. In this study, the inhibition of KSFY06 on experimental oxidative senescence was observed by analyzing the serum and tissues of mice, which laid a theoretical foundation for further research on human and industrial development.

## 2 MATERIALS AND METHODS

### 2.1 Laboratory strain

*Lactobacillus plantarum* KSFY06 is preserved in the China General Microbiological Culture Collection (CGMCC, Beijing, China) (CGMCC No. 15659). KSFY06 is separated from yoghurt in Xinjiang, China. *Lactobacillus* *delbrueckii* subsp. *bulgaricus* (LB, CGMCC No. 1.16075) as a comparative strain with KSFY06 for this study.

### 2.2 Animal models of oxidation‐induced aging

Kunming (KM) male mice, 6 weeks old, weighing 20–25 g, was purchased from the Laboratory Animal Center of Chongqing Medical University (Chongqing, China). Before the experiment, the mice were fed with drinking water freely. After 1 week of adaptive feeding, they were randomly divided into six groups, 10 mice in each group (*n* = 10/group): normal, control, vitamin C (Vc), *L. delbrueckii* subsp*. bulgaricus* (LB), KSFY06 low‐dose group (KSFY06‐L), and KSFY06 high‐dose group (KSFY06‐H). The experiment lasted for 8 weeks. Finally, all mice were fasted for 24 hr, the eyeballs were removed, blood was taken, and serum was prepared by centrifugation. Extract brain, liver, kidney, spleen, and heart. The ratio of tissue weight to the final weight of the animal is the organ index (Liu et al., 2018; Zhao, Tian, et al., 2018). Each group is treated as shown in Table 1.

**Table 1:** Group of experiment animals

Group	Treatment
Normal	Physiological saline (0.2 ml/10 g)
Control	Physiological saline (0.2 ml/10 g) + d‐galactose (120 mg/kg)
LB	LB (2.0 × 10^9^ cfu/kg, 0.2 ml/10 g) + d‐galactose (120 mg/kg)
VitC	VitC (100 mg/kg, 0.2 ml/10 g) + d‐galactose (120 mg/kg)
KSFY06‐H	KSFY06 (2.0 × 10^9^ cfu/kg, 0.2 ml/10 g) + d‐galactose (120 mg/kg)
KSFY06‐L	KSFY06 (2.0 × 10^8^ cfu/kg, 0.2 ml/10 g) + d‐galactose (120 mg/kg)

### 2.3 Activities of SOD, GSH, GSH‐Px, CAT, and MDA in the serum and liver

The blood of Kunming mice was collected, to prepare serum (730 × g, 10 min, 4°C), −80°C save. The activities of superoxide dismutase (SOD, catalog number: A001‐1), catalase (CAT, catalog number: A007‐1), glutathione (GSH, catalog number: A006‐2), glutathione peroxidase (GSH‐Px, catalog number: A005‐1), and malondialdehyde (MDA, catalog number: A003‐1) in serum and liver were determined by kit (Nanjing Jiancheng Institute of Bioengineering).

### 2.4 Pathological observation of skin, liver, and spleen tissues

The 1 cm^2^ sample of skin, liver, and spleen of each mouse was immediately fixed in 10% neutral formalin fixative for 48 hr for histopathological analysis, after H&E staining (hamatoxylin and eosin stain) (Cardiff, Miller, & Munn, 2014), histopathological observation, microscopic examination, and image acquisition and analysis (Zhao et al., 2019).

### 2.5 Quantitative PCR (qPCR) assay

About 50 mg of liver tissue and spleen tissue were homogenized, total RNA in liver tissue and spleen tissue was extracted with TRIzol, and total RNA concentration was measured by a micro spectrophotometer. Total RNA samples were used as templates to generate cDNA by reverse transcription. Then, add 10 μl Master, 0.6 μl upstream primer, 0.6 μl downstream primer, 1 μl cDNA (1 μg/μl) template, and 7.8 μl sterile ultrapure water to the octatube and mix by centrifugation. Finally, it was amplified by qPCR instrument. The amplification conditions were as follows: denaturation at 95°C for 15 min, annealing at 60°C for 1 hr, extension at 95°C for 15 min, and then 40 cycles. An internal reference was GAPDH, and the relative expression level of each gene was calculated by the 2^−ΔΔCt^ method (Zhang et al., 2018). The corresponding gene primer sequences are shown in Table 2.

**Table 2:** Sequences of primers used in the qPCR assay

Gene name	Sequence
eNOS	Forward: 5′‐TCAGCCATCACAGTGTTCCC‐3′
	Reverse: 5′‐ATAGCCCGCATAGCGTATCAG‐3′
nNOS	Forward: 5′‐ACGGCAAACTGCACAAAGC‐3′
	Reverse: 5′‐CGTTCTCTGAATACGGGTTGTTG‐3′
iNOS	Forward: 5′‐GTTCTCAGCCCAACAATACAAGA‐3′
	Reverse: 5′‐GTGGACGGGTCGATGTCAC‐3′
CAT	Forward: 5′‐GGAGGCGGGAACCCAATAG‐3′
	Reverse: 5′‐GTGTGCCATCTCGTCAGTGAA‐3′
SOD1	Forward: 5′‐AACCAGTTGTGTTGTCAGGAC‐3′
	Reverse: 5′‐CCACCATGTTTCTTAGAGTGAGG‐3′
SOD2	Forward: 5′‐CAGACCTGCCTTACGACTATGG‐3′
	Reverse: 5′‐CTCGGTGGCGTTGAGATTGTT‐3′
GAPDH	Forward: 5′‐AGGTCGGTGTGAACGGATTTG‐3′
	Reverse: 5′‐GGGGTCGTTGATGGCAACA‐3′

### 2.6 Statistical analysis

Parallel experiments were conducted three times, and the results were averaged. SPSS 22 software (IBM Corporation, North Castle, NY, USA) was used for one‐way ANOVA. The value of *p* < .05 was considered significant. All figures are drawn by Origin 8.0 software (Zhu et al., 2019).

## 3 RESULTS

### 3.1 Organ indices in mice

As shown in Table 3, the results showed that the indexes of heart, liver, spleen, and kidney in the control group were significantly lower than those in the normal group (*p* < .05), indicating that d‐galactose injection was the cause of organ aging. However, the indexes of heart, liver, spleen, and kidney in the treated group were significantly higher than those in the control group. Moreover, compared with other treatment groups, KSFY06‐H and LB showed more obvious effects on preventing organ aging.

**Table 3:** Organ index of mice in each group (*n* = 10)

Group	Liver index (mg/g)	Spleen index (mg/g)	Kidney index (mg/g)	Heart index (mg/g)
Normal	48.82 ± 0.16^e^	3.37 ± 0.05^c^	14.20 ± 0.04^e^	5.58 ± 0.02^b^
Control	39.14 ± 0.11^a^	2.09 ± 0.02^a^	10.32 ± 0.05^a^	4.68 ± 0.01^a^
LB	45.21 ± 0.12^d^	4.04 ± 0.04^d^	12.24 ± 0.06^d^	6.08 ± 0.02^c^
VitC	44.06 ± 0.06^c^	2.97 ± 0.02^b^	12.28 ± 0.04^d^	5.30 ± 0.01^b^
KSFY06‐H	45.76 ± 0.10^d^	2.84 ± 0.01^b^	12.22 ± 0.03^c^	5.37 ± 0.03^b^
KSFY06‐L	42.64 ± 0.03^b^	2.25 ± 0.01^a^	11.06 ± 0.02^b^	5.17 ± 0.04^b^

Note

Data are means ± *SD* (*n* = 10/group). ^a–e^ In the same column, different letters indicated significant differences between the two groups (*p* < .05), and the same letters indicated no significant difference between the two groups (*p* > .05) according to Duncan's multirange test. Normal = normal mice; Control = mice treated with d‐galactose injection (120 mg/kg per day); VitC = mice treated with d‐galactose and vitamin C (100 mg/kg per day); KSFY06 = mice treated with d‐galactose and increasing doses (L, H) of *Lactobacillus plantarum* KSFY06 (2 × 10^8^, 2 × 10^9^ cfu/kg per day); and LB = mice treated with d‐galactose and *Lactobacillus delbrueckii* subsp. *bulgaricus* (2 × 10^9^ cfu/kg per day).

### 3.2 Activities of SOD, GSH, GSH‐Px, CAT, and MDA

As shown in Tables 4 and 5, the MDA activity in serum and liver of the control group was the highest, and SOD, GSH, GSH‐Px, and CAT activities were the lowest. Following treatment with LB, KSFY06, or vitamin C, the activity of MDA decreased, and the activities of SOD, GSH, GSH‐Px, and CAT were increased. In particular, the indicator activities of MDA, SOD, GSH, GSH‐Px, and CAT in mice treated with the highest dose of KSFY06 were very close to normal group, illustrating that highest dose of *Lactobacillus* KSFY06 was better at delaying the aging effect than LB in mice for those aging indicators.

**Table 4:** Activities of SOD, GSH, GSH‐Px, CAT, and MDA in serum of mice (*n* = 10)

Group	SOD (U/ml)	GSH (mg/L)	GSH‐Px (U/ml)	CAT (U/ml)	MDA (nmol/ml)
Normal	76.38 ± 0.56^c^	3.55 ± 0.12^c^	394.74 ± 23.12^d^	4.72 ± 0.26^e^	5.34 ± 0.95^a^
Control	71.88 ± 0.38^a^	1.71 ± 0.22^a^	294.74 ± 15.21^a^	2.89 ± 0.06^a^	20.97 ± 0.81^e^
LB	73.88 ± 0.72^ab^	3.02 ± 0.31^bc^	351.32 ± 10.09^b^	3.12 ± 0.39^b^	9.60 ± 1.64^d^
VitC	74.35 ± 0.44^ab^	2.81 ± 0.45^b^	309.87 ± 20.78^d^	3.29 ± 0.28^c^	8.56 ± 0.22^c^
KSFY06‐H	75.43 ± 0.61^bc^	3.84 ± 0.27^d^	369.47 ± 27.23^e^	4.92 ± 0.19^f^	5.44 ± 0.77^ab^
KSFY06‐L	72.81 ± 0.31^a^	2.96 ± 0.15^b^	315.79 ± 30.45^c^	3.51 ± 0.13^d^	6.65 ± 1.20^d^

Note

Data are means ± *SD* (*n* = 10/group). ^a–e^ In the same column, different letters indicated significant differences between the two groups (*p* < .05), and the same letters indicated no significant difference between the two groups (*p* > .05) according to Duncan's multirange test. Normal = normal mice; Control = mice treated with d‐galactose injection (120 mg/kg per day); VitC = mice treated with d‐galactose and vitamin C (100 mg/kg per day); KSFY06 = mice treated with d‐galactose and increasing doses (L, H) of *Lactobacillus plantarum* KSFY06 (2 × 10^8^, 2 × 10^9^ cfu/kg per day); and LB = mice treated with d‐galactose and *Lactobacillus delbrueckii* subsp. *bulgaricus* (2 × 10^9^ cfu/kg per day).

**Table 5:** Activities of SOD, GSH, GSH‐Px, CAT, and MDA in liver of mice (*n* = 10)

Group	SOD (U/mg prot)	GSH (mg/g prot)	GSH‐Px (U/mg prot)	CAT (U/mg prot)	MDA (nmol/mg prot)
Normal	245.42 ± 23.33^d^	66.44 ± 11.07^e^	1,129.11 ± 79.07^e^	145.92 ± 12.22^b^	77.13 ± 7.64^d^
Control	169.45 ± 4.09^b^	35.07 ± 4.01^ab^	882.63 ± 80.02^a^	102.50 ± 7.45^e^	141.52 ± 16.88^a^
LB	182.24 ± 15.75^b^	33.12 ± 2.09^a^	975.35 ± 94.35^bc^	108.13 ± 17.74^a^	60.40 ± 10.46^ab^
VitC	242.40 ± 12.81^cd^	46.16 ± 2.78^cd^	1,070.42 ± 66.58^d^	125.73 ± 4.83^bc^	85.78 ± 7.27^c^
KSFY06‐H	227.68 ± 22.27^c^	50.63 ± 7.23^d^	1,010.95 ± 96.03^cd^	124.66 ± 19.24^cd^	97.52 ± 7.78^c^
KSFY06‐L	144.69 ± 19.30^a^	39.72 ± 5.64^bc^	902.45 ± 53.63^ab^	115.07 ± 12.86^d^	96.06 ± 12.19^b^

Note

Data are means ± *SD* (*n* = 10/group). ^a–e^ In the same column, different letters indicated significant differences between the two groups (*p* < .05), and the same letters indicated no significant difference between the two groups (*p* > .05) according to Duncan's multirange test. Normal = normal mice; Control = mice treated with d‐galactose injection (120 mg/kg per day); VitC = mice treated with d‐galactose and vitamin C (100 mg/kg per day); KSFY06 = mice treated with d‐galactose and increasing doses (L, H) of *Lactobacillus plantarum* KSFY06 (2 × 10^8^, 2 × 10^9^ cfu/kg per day); and LB = mice treated with d‐galactose and *Lactobacillus delbrueckii* subsp. *bulgaricus* (2 × 10^9^ cfu/kg per day).

### 3.3 Pathological observation of mouse liver, spleen, and skin

**Figure 1:**
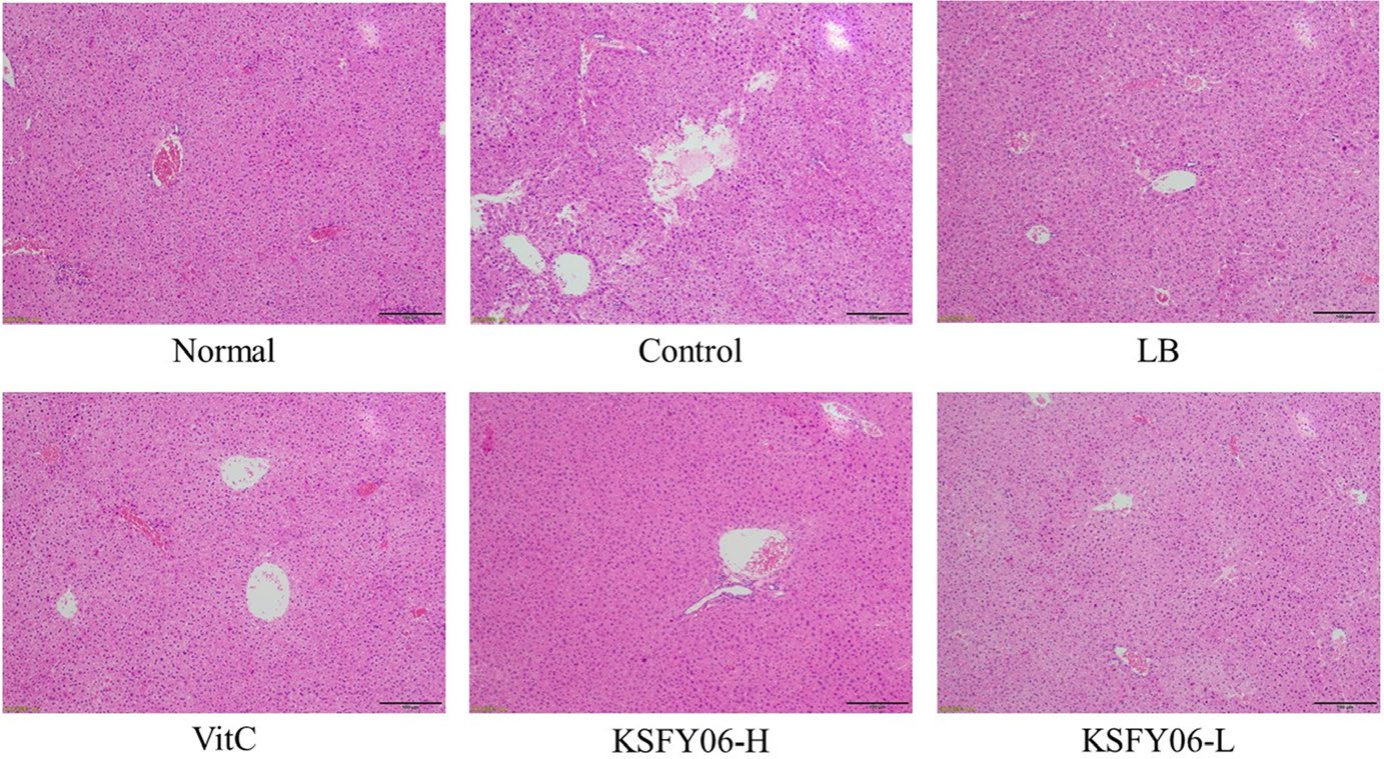
H&E pathological observation of liver in mice. Normal = normal mice; Control = mice treated with d‐galactose injection (120 mg/kg per day); VitC = mice treated with d‐galactose and vitamin C (100 mg/kg per day); KSFY06 = mice treated with d‐galactose and increasing doses (L, H) of *Lactobacillus plantarum* KSFY06 (2 × 10^8^, 2 × 10^9^ cfu/kg per day); and LB = mice treated with d‐galactose and *Lactobacillus delbrueckii* subsp. *bulgaricus* (2 × 10^9^ cfu/kg per day)

The histological micrographs of the liver are shown in Figure 1. In the normal group, the liver cell morphology was regular, the size and staining were uniform, there were no signs of inflammation, the hepatic cell line was arranged in order, the boundary was clear, and the central vein was distributed radially. However, compared with mice of the normal group, mice in the control group contained hepatocytes that were disorganized and showed irregular morphology, loss of cell boundary, an irregular central vein shape, cell swelling, and widespread signs of inflammatory infiltration. Compared with mice of the control group, those treated with vitamin C, LB, or KSFY06 showed a reduction in this chemical damage. In the KSFY06‐treated groups, liver damage was less severe than in controls (only d‐galactose treatment), and thus, the efficacy of KSFY06 in preventing liver atrophy was better. Moreover, the effect of protecting the liver is related to the dose.

**Figure 2:**
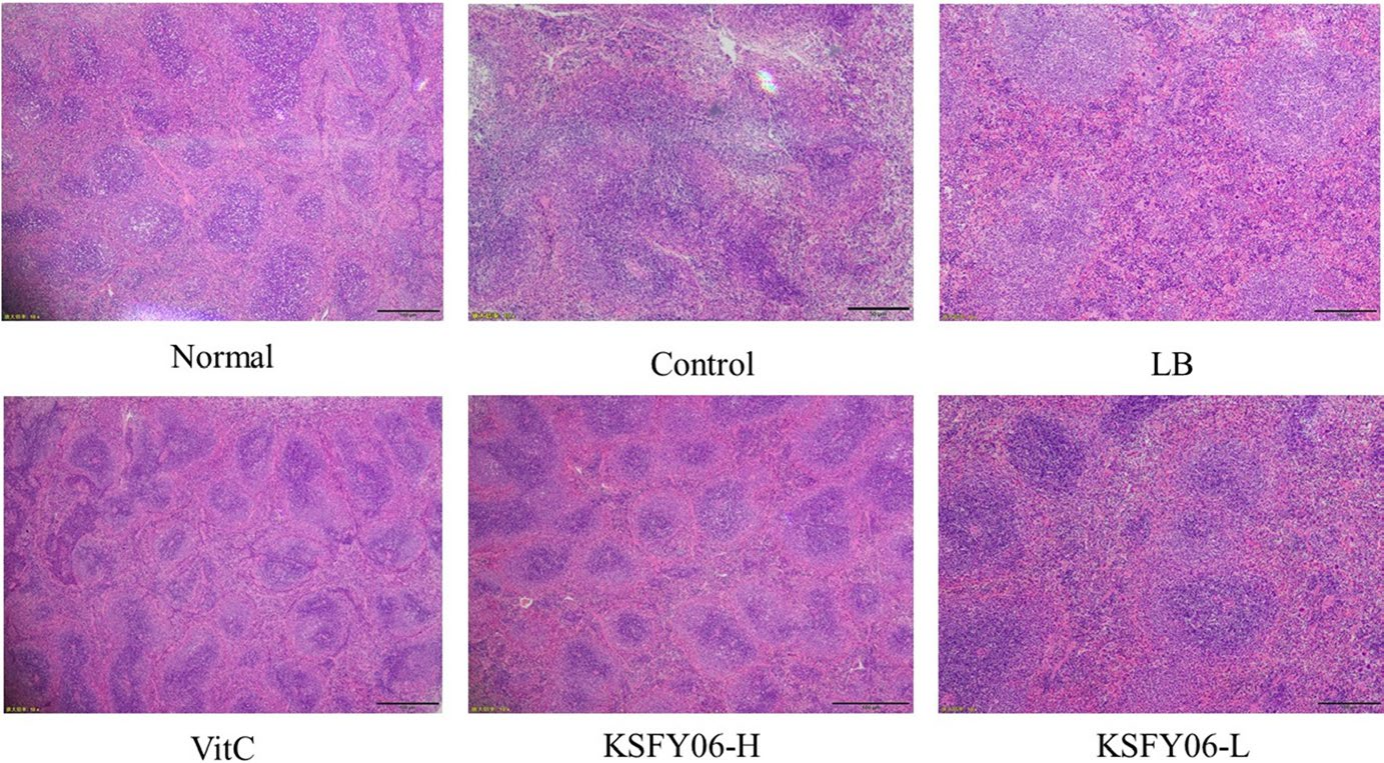
H&E pathological observation of spleen in mice. Normal = normal mice; Control = mice treated with d‐galactose injection (120 mg/kg per day); VitC = mice treated with d‐galactose and vitamin C (100 mg/kg per day); KSFY06 = mice treated with d‐galactose and increasing doses (L, H) of *Lactobacillus plantarum* KSFY06 (2 × 10^8^, 2 × 10^9^ cfu/kg per day); and LB = mice treated with d‐galactose and *Lactobacillus delbrueckii* subsp. *bulgaricus* (2 × 10^9^ cfu/kg per day)

Figure 2 is a histological micrograph of the spleen. The spleen envelope was flat, the spleen trabecular structure was normal, and the boundary between the red pulp and the white pulp was clear in the normal group mice. In the control group, the spleen tissue was irregular in morphology, red blood cells and white pulp lymphocytes were reduced, red pulp cord was narrowed, and cell density was decreased. After treatment with vitamin C, LB, and KSFY06, the spleen tissue morphology was significantly improved in the treatment group. The red and white pulp boundaries were clear, a flat spleen capsule, and the cells are arranged neatly and tightly. The treatment groups showed similar micrographs to those of the normal group mice, with KSFY06 showing the best effects.

**Figure 3:**
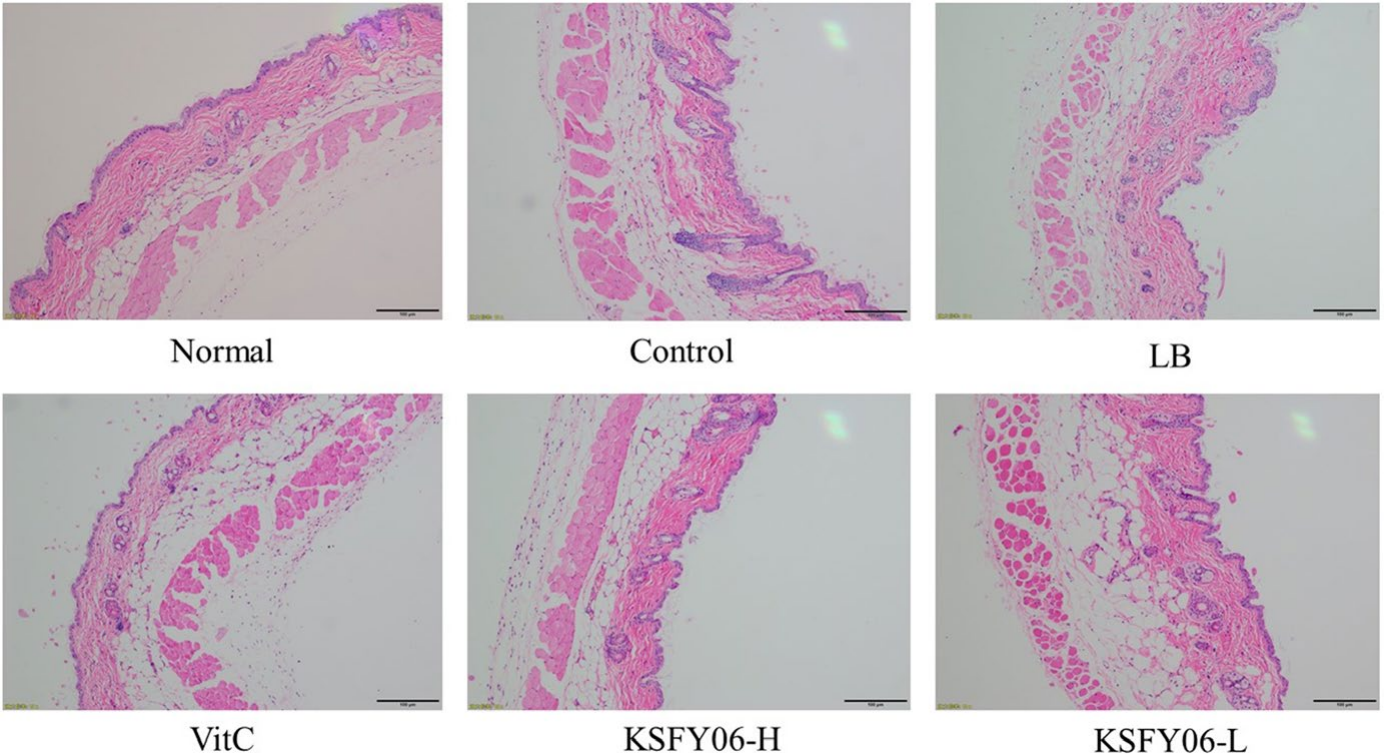
H&E pathological observation of skin in mice. Normal = normal mice; Control = mice treated with d‐galactose injection (120 mg/kg per day); VitC = mice treated with d‐galactose and vitamin C (100 mg/kg per day); KSFY06 = mice treated with d‐galactose and increasing doses (L, H) of *Lactobacillus plantarum* KSFY06 (2 × 10^8^, 2 × 10^9^ cfu/kg per day); and LB = mice treated with d‐galactose and *Lactobacillus delbrueckii* subsp. *bulgaricus* (2 × 10^9^ cfu/kg per day)

Figure 3 is a histological micrograph of the skin. The epidermal structure of normal skin tissue was clear and intact. The collagen fibers in the dermis are rich in content. The boundaries of the dermis and epidermis are clear and intact. The collagen fibers in the dermis are rich in content, and the number of fat vacuoles is normal. Compared with mice of the normal group, mice in the control group had a decreased collagen fiber content and increased numbers of lipid vacuoles, with endosmosis occurring in the dermis and a blurred boundary between the dermis and epidermis. Compared with mice of the control group, treatment with vitamin C, LB, or KSFY06 significantly improved the skin tissue lipid vacuoles, endosmosis, and the boundary between the epidermis and dermis, alleviating skin lesions in the d‐galactose‐induced aging mice. Moreover, mice in the KSFY06‐H group had micrographs closer to the normal mice than those in LB and Vc groups, and thus, the efficacy of KSFY06 in preventing skin lesions was better.

### 3.4 Gene expression content of mouse liver

**Figure 4:**
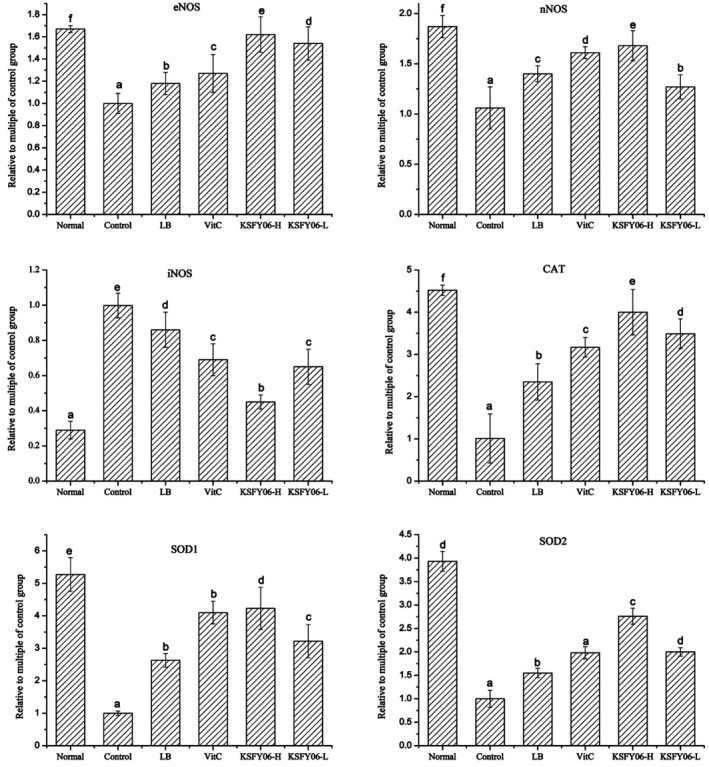
mRNA expression level of SOD1, SOD2, CAT, eNOS, nNOS, and iNOS in liver tissue of mice. ^a–f^ In the same column, different letters indicated significant differences between the two groups (*p* < .05), and the same letters indicated no significant difference between the two groups (*p* > .05) according to Duncan's multirange test. Normal = normal mice; Control = mice treated with d‐galactose injection (120 mg/kg per day); VitC = mice treated with d‐galactose and vitamin C (100 mg/kg per day); KSFY06 = mice treated with d‐galactose and increasing doses (L, H) of *Lactobacillus plantarum* KSFY06 (2 × 10^8^, 2 × 10^9^ cfu/kg per day); and LB = mice treated with d‐galactose and *Lactobacillus delbrueckii* subsp. *bulgaricus* (2 × 10^9^ cfu/kg per day)

The expression of SOD1, SOD2, CAT, eNOS, nNOS, and iNOS in liver tissues of mice was determined by qPCR. The results are shown in Figure 4. The expression levels of SOD1, SOD2, CAT, eNOS, and nNOS were the highest in the liver tissues of normal mice, while the expression level of iNOS was the lowest. However, the mRNA expression levels of the above genes in the control group were reversed. After vitamin C, LB, or KSFY06 treatment, the expression of SOD1, SOD2, CAT, eNOS, nNOS, and iNOS in aging mice was significantly improved (*p* < .05), with high dose of KSFY06 showing the best effects.

### 3.5 Gene expression content of mouse spleen

**Figure 5:**
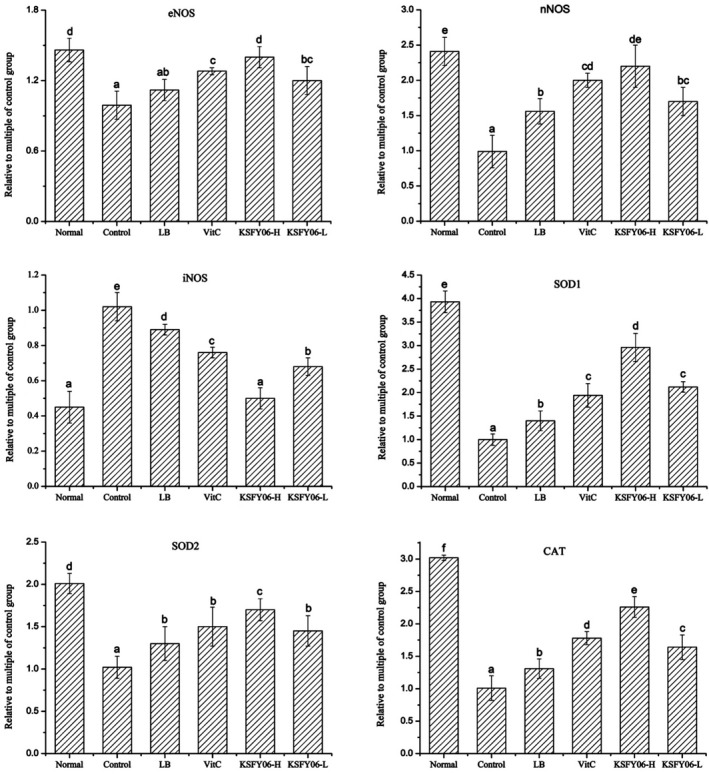
mRNA expression level of SOD1, SOD2, CAT, eNOS, nNOS, and iNOS in spleen tissue of mice. ^a–e^ In the same column, different letters indicated significant differences between the two groups (*p* < .05), and the same letters indicated no significant difference between the two groups (*p* > .05) according to Duncan's multirange test. Normal = normal mice; Control = mice treated with d‐galactose injection (120 mg/kg per day); VitC = mice treated with d‐galactose and vitamin C (100 mg/kg per day); KSFY06 = mice treated with d‐galactose and increasing doses (L, H) of *Lactobacillus plantarum* KSFY06 (2 × 10^8^, 2 × 10^9^ cfu/kg per day); and LB = mice treated with d‐galactose and *Lactobacillus delbrueckii* subsp. *bulgaricus* (2 × 10^9^ cfu/kg per day)

The gene expression of mouse spleen tissue is shown in Figure 5. Compared with the normal group, the mRNA expression of SOD1, SOD2, CAT, eNOS, and nNOS in the spleen tissue of the aging control group was the lowest, and the mNA expression level of iNOS was the highest. After treatment with vitamin C, LB, or KSFY06, the mRNA expression of SOD1, SOD2, CAT, eNOS, and nNOS was increased, and the mNA expression of iNOS was decreased. The expression of KSFY06 high‐dose treatment group was close to that of normal mice, which was stronger than that of vitamin C and LB‐treated mice.

## 4 DISCUSSION

Lactic acid bacteria have special physiological functions through organic acids, special enzymes, acid bacteria, and other substances produced by fermentation. They are widely used in the food industry and livestock and poultry, and have a great impact on people's daily life (Landete et al., 2017). Lactic acid bacteria strains isolated from fermented foods may have antioxidant activity (Kenfack et al., 2018; Leite et al., 2015; Tang, Xing, Li, Wang, & Wang, 2017). Thus, we focused on studying the antioxidant activity of the KSFY06 strain isolated from yogurt. The continuous subcutaneous injection of d‐galactose has been shown to induce aging in model mice, leading to an increase in the active oxygen free radicals (ROS) in the mice and a decrease in the antioxidant enzyme activity, ultimately leading to an increase in oxidative stress and accelerating the aging processes (Zhang, Li, Cui, & Zuo, 2005). The theory of aging and self‐sufficiency suggests that the increased free radicals may lead to the degradation of the aging‐related mechanisms. Therefore, we used the d‐galactose‐induced oxidative stress and aging model mice to explore the antioxidation and antiaging effects of the *L. plantarum* KSFY06.

Organ indices of mice are basic indicators and an important basis for biomedical research. The aging of the human body and liver atrophy are most obvious because the liver is most vulnerable to ROS attack (Xu et al., 2016). The liver is also an immune organ in animals, and liver aging is the leading cause of immune aging (Knolle & Gerken, 2000)**.** Studies have shown that the liver index of aging mice is significantly lower than that of normal nonaging mice (Sang et al., 2017). The kidney is the metabolic organ of mice, and its weight loss has a great impact on metabolic capacity. The spleen is closely related to the cellular immune response of animals, but the immune ability of aging mice is relatively weak. A decrease in the spleen weight indicates the occurrence of organ atrophy, which can reduce the immune function of the animal (Xu et al., 2018). Therefore, changes in the structure and function of mouse organs can be observed by organ index and are important reference values for evaluating whether construction of a mouse model of aging is successful (Bonthius, Winters, Karacay, Bousquet, & Bonthius, 2015). The results of this study show that d‐galactose can cause oxidative aging in rats and atrophy of organs. After treatment with *L. plantarum* KSFY06 (2 × 10^9^ cfu/kg), aging was effectively delayed, suggesting the pronounced antiaging effect of KSFY06.

Pathological observation is the observation of changes in the morphology of organs, tissues, or cells in order to determine the pathological changes of the body (Di Meo, Reed, Venditti, & Victor, 2016). Skin, spleen, and liver can reflect some of the characteristic changes that occur in the body due to aging (Qian et al., 2018). According to this study, compared with the mice in normal group, the liver and spleen tissues of the aging group were atrophied and the skin was damaged. After vitamin C, LB, or KSFY06 treatment, the abnormal morphology of the tissues was significantly improved, and the improvement effect of KSFY06 was the most obvious. Morphological tissue of KSFY06‐treated mice is closest to the normal group.

In the biological oxidation process of the body, a large amount of free radicals are produced. The balance of free radicals in the body is accomplished by a number of antioxidant defense systems, including GSH‐Px, SOD, CAT, reduced GSH, and vitamin C. Their synergistic effect converts excess free radicals in the body into oxygen molecules and water molecules, thereby acting as an antioxidant (Zhang et al., 2008). Lactic acid bacteria have antioxidant activity and have the function of scavenging free radicals, which can assist the antioxidant enzymes to complete the antioxidant effect. In addition, the bacteria release antioxidant enzymes such as SOD during the metabolism in the body to block the oxidation process (Lobo, Patil, Phatak, & Chandra, 2010).

Clinical studies have shown that changes occur in SOD, GSH‐Px, GSH, CAT, and MDA activities in patients treated for antioxidation (Zhao et al., 2017). SOD protects the cells from damage caused by oxidation, indirectly reflecting their antioxidant activity (Feng et al., 2016). GSH‐Px removes the lipid peroxidation induced by active oxygen and hydroxyl free radicals and protects the integrity of the cell membrane structure and function (Zhuang, Ma, Guo, & Sun, 2017). In this study, the *L. plantarum* KSFY06 significantly increases the SOD activity (Table 4) and GSH‐Px activity (Table 5) in the serum and liver tissue of the mouse. GSH also has an antioxidant effect, and GSH reduces hydrogen peroxide produced by glutathione peroxidase (GSH‐PX). The experimental results show that the injection of d‐galactose causes severe cellular oxidative stress, resulting in a decrease in GSH activity in liver tissues. In addition, CAT activity was also significantly reduced, indicating a decrease in total antioxidant capacity in model mice. In this research, *L. plantarum* KSFY06, *Lactobacillus bulgaricus* LB, and vitamin C were shown to inhibit the decrease of GSH, GSH‐Px and CAT activities in liver and serum. From the mouse liver tissue measurement indicators GSH‐Px and CAT (Table 5), vitamin C was as good as KSFY06‐H in the antioxidant effect. The antioxidant effect of *L. plantarum* KSFY06 was also related to the dose, and the high dose is more obvious than the low‐dose antioxidant effect. Wa et al. (2019) found that probiotics significantly improved the antioxidant capacity of hyperlipidemic mice and increased the activity of antioxidant enzymes including SOD, GSH‐PX, GPT, and GOT. Therefore, it is further proved that LAB can enhance the synthesis of antioxidant enzymes. MDA is an oxidation end product of free radicals acting on lipid peroxidation (Zhao, Tian, et al., 2018; Zhao, Song, et al., 2018a), which affects the activity of key enzymes in mitochondrial respiratory chain complex and mitochondria in vitro, and can also aggravate membrane damage. Therefore, the amount of malondialdehyde can reflect the degree of lipid peroxidation in the body. Malondialdehyde also can indirectly reflect the degree of cell damage (Hosen, Islam, Begum, Kabir, & Howlader, 2015). This study found that KSFY06 can significantly reduce the activity of malondialdehyde in liver tissue of mice. The malondialdehyde activity in liver tissue of high‐dose KSFY06 mice is close to normal level. Other in vivo studies have shown that MDA activity in serum of hyperlipidemic mice fed with *Lactobacillus canis* is reduced (Wang, Xie, et al., 2016; Wang, Zhou, et al., 2016). Qian et al. (2018) found that MDA activity in the serum and liver of antioxidized mice was decreased though *L. plantarum* CQPC‐11 was administered. This study has shown that *L. plantarum* KSFY06 can reduce the MDA activity in the serum and liver (Tables 4 and 5) and further demonstrates that KSFY06 can enhance the antioxidative defense system of the organism. Our study further confirms that KSFY06 can delay the aging of mice by regulating SOD, GSH‐px, GSH, CAT, and MDA activities.

To further elucidate the antioxidant mechanism, this study has examined the changes in mRNA expression levels of eNOS, nNOS, iNOS, CAT, SOD1, and SOD2. NO is a highly unstable biological free radical. It is fat‐soluble, free to cross the cell membrane and highly oxidizing, with a biological half‐life of only 3–5 s. Its production depends on nitric oxide synthase (NOS), including nNOS, eNOS, and iNOS, and it has a very important biological role in regulating several systems, including the heart, cerebrovascular functions, and immunity (Eroglu et al., 2017). Under physiological conditions, NO plays an important biological role in heart, cerebral vascular regulation, nerve, immune regulation, etc., but in the case of excessive NO, it causes cell damage (Dong, Zheng, Lu, & Yang, 2017). iNOS is one of the kinases associated with the pathogenesis of Alzheimer's disease. Excessive release of high concentrations of NO leads to oxidative damage (Kalantar‐Zadeh & Fouque, 2017). nNOS and eNOS are basal‐expressing tissue‐constituting enzymes that participate in the entire process of learning and memory impairment (Bonthius et al., 2015). The content of nNOS and eNOS decreases after oxidative damage in the body, while increasing the levels of nNOS and eNOS in the body will also effectively control aging (Bonthius et al., 2015). In this study, *L. plantarum* KSFY06 can also effectively control the expression of iNOS in mouse liver tissue and spleen tissue, while increasing the expression of nNOS and eNOS to slow down the aging.

In animals, SOD mainly exists in blood as cytosolic SOD1, while SOD2 is distributed in the mitochondrial matrix, which can control free radicals to keep them at low activities and maintain good health (Zhao, Tian, et al., 2018; Zhao, Song, et al., 2018a). Studies have shown that the decreased expression of SOD2 is usually caused by tissue atrophy. Oxidative aging or chemical aging can also significantly reduce the activity of SOD1 (Hart et al., 2015). CAT has the effect of scavenging oxygen free radicals and promoting the decomposition of hydrogen peroxide, avoiding oxidation injury to cells in the body (Selvaratnam & Robaire, 2016). From the experimental results, the SOD1, SOD2 mRNA expression levels, and CAT in the liver tissues of the aging control group were significantly decreased (*p* < .05), indicating that intraperitoneal injection of d‐galactose could cause aging in mice. However, the mRNA expression levels of SOD1, SOD2, and CAT in liver tissue and spleen of mice were significantly increased after treatment with *L. plantarum* KSFY06 (*p* < .05). All experimental results show that *L. plantarum* KSFY06 can inhibit the oxidative senescence induced by d‐galactose in mice by increasing the activities of SOD1, SOD2, and CAT in the body.

## CONFLICT OF INTEREST

The authors of this manuscript state that they do not have conflict of interest to declare.

## ETHICAL APPROVAL

The protocol for these experiments was approved by the Animal Ethics Committee of Chongqing Collaborative Innovation Center for Functional Food (201812007B).
